# Dietary patterns and cardiorespiratory fitness in midlife and subsequent all-cause dementia: findings from the Cooper Center Longitudinal Study

**DOI:** 10.1186/s12966-024-01663-x

**Published:** 2024-09-27

**Authors:** Clare Meernik, Sigal Eilat-Adar, David Leonard, Carolyn E. Barlow, Yariv Gerber, Riki Tesler, Carmen Byker Shanks, Kelley Pettee Gabriel, Andjelka Pavlovic, Laura F. DeFina, Kerem Shuval

**Affiliations:** 1 The Cooper Institute, 12330 Preston Rd, Dallas, TX 75230 USA; 2 Levinsky-Wingate Academic College, Netanya, Israel; 3 School of Public Health, Faculty of Medicine, Tel Aviv University, Tel Aviv, Israel; 4 Department of Health Systems Management, Ariel University, Ariel, Israel; 5 Gretchen Swanson Center for Nutrition, Omaha, NE USA; 6 Department of Epidemiology, University of Alabama at Birmingham, Birmingham, AL USA

**Keywords:** Mediterranean diet, DASH diet, Cardiorespiratory fitness, Dementia, Alzheimer’s disease

## Abstract

**Background:**

Identifying lifestyle factors that independently or jointly lower dementia risk is a public health priority given the limited treatment options available to patients. In this cohort study, we examined the associations between Mediterranean or Dietary Approaches to Stop Hypertension (DASH) diet adherence and cardiorespiratory fitness (CRF) with later-life dementia, and assessed whether the associations between dietary pattern and dementia are modified by CRF.

**Methods:**

Data are from 9,095 adults seeking preventive care at the Cooper Clinic (1987–1999) who completed a 3-day dietary record and a maximal exercise test. Alzheimer’s disease and related disorders or senile dementia (i.e., all-cause dementia) was identified from Medicare administrative claims (1999–2019). Illness-death models were used to estimate hazard ratios (HRs) and 95% confidence intervals (CIs) for the associations between Mediterranean or DASH diet adherence (primary exposure), CRF (secondary exposure), and all-cause dementia, adjusted for demographic and clinical factors. An interaction term was included between diet score and CRF to assess effect modification by CRF.

**Results:**

The mean age at baseline was 50.6 (standard deviation [SD]: 8.4) years, and a majority of the study sample were men (77.5%) and White (96.4%). 1449 cases of all-cause dementia were identified over a mean follow-up of 9.2 (SD: 5.8) years. Neither Mediterranean nor DASH diet adherence was associated with dementia risk in fully adjusted models (HR per SD of Mediterranean diet score: 1.00, 95% CI: 0.94, 1.05; HR per SD of DASH diet score: 1.02, 95% CI: 0.96, 1.08). However, participants with higher CRF had a decreased hazard of dementia (HR, per metabolic equivalent of task [MET] increase, Mediterranean model: 0.95, 95% CI: 0.92, 0.98; HR, per MET increase, DASH model: 0.96, 95% CI: 0.92, 0.97). No effect modification by CRF was observed in the association between diet and dementia.

**Conclusions:**

In this sample of apparently healthy middle-aged adults seeking preventive care, higher CRF at midlife was associated with a lower risk of all-cause dementia, though adherence to a Mediterranean or DASH diet was not, and CRF did not modify the diet-dementia association. CRF should be emphasized in multimodal interventions for dementia prevention and investigated among diverse samples.

**Supplementary Information:**

The online version contains supplementary material available at 10.1186/s12966-024-01663-x.

## Background

Alzheimer’s disease and other dementias have no cure and limited treatment options are available for the estimated 6.7 million Americans ages ≥ 65 years living with this group of diseases [1, 2], highlighting the public health importance of risk reduction through modifiable lifestyle factors [3, 4]. While age, genetics, and a family history of dementia are the strongest risk factors for dementia [1], modifiable risk factors may prevent or delay up to 40% of dementia cases [5].

In this study, we were primarily interested in the relation between two specific dietary patterns—the Mediterranean diet and the Dietary Approaches to Stop Hypertension (DASH) diet—and dementia risk. The Mediterranean and DASH diets both encourage increased consumption of fruits, vegetables, whole grains, and legumes, while limiting processed foods and red meat; the Mediterranean diet additionally promotes fiber and DASH limits sodium [6, 7]. While both diets reduce the risks for multiple chronic diseases [8, 9], the evidence in relation to cognitive outcomes is mixed [10, 11]. For instance, in one recent meta-analysis of cohort studies in older adults (aged ≥ 60 years), higher adherence to the Mediterranean diet was associated with less decline in global cognition but had no association with mild cognitive impairment (MCI) or dementia [12]. Conversely, another recent meta-analysis with similar inclusion criteria found that the highest adherence to the Mediterranean diet was associated with reduced all-cause dementia (including MCI) [13]. Meanwhile, high adherence to the DASH diet was associated with 15% reduction in neurodegenerative diseases (combined outcome of Parkinson’s disease and cognitive impairment) in a meta-analysis of seven studies [9]. Other comprehensive reviews published within the past five years have generally concluded that adherence to the Mediterranean or DASH diet is associated with slower cognitive decline and a lower risk of Alzheimer’s disease, but the evidence remains limited in relation to all-cause dementia [8, 14–16].

We were secondarily interested in cardiorespiratory fitness (CRF) given evidence that higher CRF may be protective of dementia [17–21]. However, previous studies—including within our study cohort—have not accounted for dietary intake (a potential confounder) in examining associations between CRF and dementia. Additionally, given the correlation between CRF and dietary patterns [22], we were interested in exploring the potential interaction between these two lifestyle factors, which may inform more effective, multimodal prevention strategies [23]. Further, studies to date investigating diet in relation to dementia risk have generally only considered self-reported physical activity (primarily as a confounder); none have examined CRF specifically or its interaction with diet [15, 24]. Self-reported physical activity is associated with a lower risk of dementia [4, 25–27], but it can be prone to measurement error [28] and is a worse predictor of morbidity than CRF [29]. The potential synergistic neuroprotective effect between a clinic-based measurement of CRF and dietary patterns has not been established and is crucial in understanding how to better prevent, delay, and manage dementia [1, 15, 23, 30, 31].

We had the following two objectives in this cohort study: (1) Examine the association between Mediterranean or DASH diet adherence at midlife, CRF at midlife, and subsequent all-cause dementia; and (2) Assess effect modification between diet and CRF in relation to all-cause dementia.

## Methods

### Study population

We examined the relationship between dietary pattern, CRF, and subsequent all-cause dementia among adults enrolled in the Cooper Center Longitudinal Study (CCLS) during 1987–1999 and whose baseline data was linked to Medicare administrative claims during 1999–2019. The CCLS is comprised of patients who visit the Cooper Clinic (Dallas, Texas) for preventive medicine examinations and provide written informed consent to participate in the study. CCLS participants complete extensive medical questionnaires and undergo laboratory testing and fitness assessment. The CCLS is approved annually by the Institutional Review Board at The Cooper Institute.

The study cohort initially comprised 9,859 participants in the CCLS who completed a baseline 3-day dietary record in 1987–1999 (the only time period of the CCLS during which 3-day dietary records were collected for research) and who were enrolled in Medicare fee-for-service (Parts A and B) during 1999–2019. We excluded participants with prevalent dementia prior to our Medicare surveillance period (*n* = 49), who were those with an earliest indication of dementia in the Medicare Chronic Conditions Warehouse (CCW) [32] prior to our surveillance period. We also excluded participants who were missing covariate data (*n* = 501); those < 20 years old (*n* = 4); those who reported a total caloric intake < 500 or > 5,000 kilocalories per day (*n* = 18); those with a body mass index (BMI) < 18.5 kg/m^2^, as it may be indicative of pre-existing illness (*n* = 66); and those with a history of myocardial infarction (*n* = 97) or stroke (*n* = 29) at baseline. The final cohort included 9,095 apparently healthy men and women.

### Dietary patterns

The primary exposures of interest were level of adherence to either a Mediterranean or DASH diet. Mediterranean and DASH diet scores were calculated from 3-day dietary records completed by participants just prior to their baseline clinic visit, as described previously [33, 34]. Briefly, after detailed instruction, participants recorded their food intake on two weekdays and one weekend day. Nutrient analysis was performed with the Food Intake Analysis System (University of Texas-Houston School of Public Health), which used the U.S. Department of Agriculture (USDA) Survey Nutrient Database [35] and the USDA Pyramid Servings database [36] to describe nutrient intake and servings data for participants.

Based on 3-day dietary record analysis, participants were first categorized into quintiles of total caloric intake. Mediterranean and DASH diet scores were then calculated within these quintiles, which ensured that diet scores were energy-adjusted, as done in a previous study [37]. Mediterranean diet score was calculated using a 10-point scale (0–9), with higher scores indicative of higher adherence to the diet [38]. Participants received a value of 1 for above median values of favorable components (vegetables except potatoes, fruits and nuts, legumes, grains, fish, mono-unsaturated/saturated fat ratio); below median values of unfavorable components (dairy, meat); or moderate consumption of alcohol (men: 10–50 g/day; women: 5–25 g/day) [38]. Otherwise, participants received a value of 0 for that particular component. Component scores were summed to obtain an overall Mediterranean diet score.

DASH diet score was calculated using a scale ranging from 8 to 40, with higher scores reflective of higher adherence to the diet [39]. Within each quintile of total energy intake, participants were categorized into quintiles of intake for each of the eight broad components of the diet. For beneficial components (fruits, vegetables, nuts and legumes, dairy, whole grains), quintile 1 (lowest intake) was assigned 1 point and quintile 5 (highest intake) was assigned 5 points; for detrimental components (sodium, red or processed meats, sugars), scoring was reversed such that participants with the lowest intake of that component received 5 points [39]. Component scores were summed to obtain an overall DASH diet score.

### Cardiorespiratory fitness (CRF)

The secondary exposure of interest was CRF (expressed as metabolic equivalents of task [METs] at the maximal workload achieved), which was estimated using a modified Balke protocol; [40] this method is highly correlated with measured maximal oxygen uptake, or VO_2max_, in men (*r* = 0.92) and women (*r* = 0.94) [41, 42]. CRF was estimated from the final treadmill speed and grade [43] and converted to METs (1 MET = 3.5 mL O_2_ uptake/kg/minute).

### All-cause dementia

The outcome of interest was Alzheimer’s disease and related disorders or senile dementia (i.e., all-cause dementia), which was identified through the Medicare CCW [32]. The CCW contains a set of chronic conditions defined by the Centers for Medicare and Medicaid Services through validated algorithms. The algorithm for all-cause dementia is described in Appendix Table 1 [32].

### Covariates

Participant characteristics were assessed at the time of baseline clinic visit. The following covariates were identified a priori for inclusion in multivariable models: age (continuous), sex (male/female), education (Bachelor’s degree or higher: yes/no), current smoking (yes/no), total daily caloric intake (kilocalories), fasting glucose (mg/dL), total cholesterol (mg/dL), resting systolic blood pressure (mm Hg), and BMI (kg/m^2^). Participants who reported a Bachelor’s degree or higher, or reported ≥ 16 years of education were categorized as having at least a Bachelor’s degree. Total daily caloric intake was based on the 3-day dietary records [33, 34]. Glucose and cholesterol profiles were assessed after a 12-hour fast. Blood pressure assessment adhered to an established protocol at the Cooper Clinic.

### Statistical analysis

Descriptive statistics of participant demographic and clinical characteristics are presented for the overall cohort and by adherence to the Mediterranean or DASH diet. Jonckheere-Terpstra statistics were used to assess trends in characteristics by diet score. Illness-death models were used to estimate hazard ratios (HRs) and 95% confidence intervals (CIs) for the associations between midlife Mediterranean or DASH diet score (primary exposure), CRF (secondary exposure), and subsequent risk of all-cause dementia. Illness-death models acknowledge the semi-competing nature of dementia and death events and incorporate a shared frailty effect within participants such that dementia and death are explicitly correlated within the model; resulting HRs estimate the risk of death without dementia, risk of dementia, and risk of death after dementia [44]. The models assume Gompertz distributed mortality and gamma distributed frailty. To enhance comparability in interpretation given differing diet score scales, we report HRs per standard deviation (SD) of diet score.

Estimated HRs were adjusted in five sequential models: (1) age, sex, clinic examination year; (2) model 1 with current smoking and education; (3) model 2 with CRF; (4) model 3 with total caloric intake and BMI; and (5) model 4 with fasting glucose, total cholesterol, and resting systolic blood pressure (i.e., cardiovascular disease [CVD] risk factors). The maximum likelihood method [45] was used to include observations with missing data on education. Total caloric intake, BMI, and CVD risk factors were hypothesized to potentially lie on the causal path between diet and dementia [4, 46], so were included last in the adjustment sequence.

We assessed effect modification by CRF by including an interaction term between diet score and CRF in a separate model adjusted for all other covariates. We conducted similar assessment for effect modification by sex. We tested for departures from proportional hazards by fitting separate models with an interaction term between diet score and age during follow-up. All analyses were conducted in SAS/STAT version 9.4 (SAS Institute, Inc., Cary, NC). The STROBE reporting guidelines were followed (Supplementary Material 1).

### Sensitivity analysis

We conducted multiple sensitivity analyses to account for alternative analytic strategies. (1) We adjusted for total energy intake using the residual method, where calorie-adjusted nutrient intakes were calculated as the residuals from regressing intake of the nutrient of interest on total caloric intake [47]. Calorie-adjusted intakes for each dietary component were then used to calculate the rankings for diet scores, as previously described. (2) We refit the primary model sequences but excluded education to ensure that results were robust to treatment of missing data on education. (3) We used a Chebyshev polynomial expansion [48] to assess non-linear trends in diet score, and subsequently analyzed diet scores as quintiles. (4) The DASH diet score specifies “low-fat” dairy as a beneficial component of the diet [39]. However, our data only allowed for the identification of dairy consumption broadly. To address this, we assessed an extreme scenario where all dairy was assumed to be high-fat in the DASH analysis, and thus all participants were deemed to be at the lowest adherence for this component (assigned 1 of 5 possible points). (5) To address potential reverse causality, we excluded participants (*n* = 44) with an earliest indication of dementia at ages ≤ 67 years (i.e., two years after Medicare eligibility). (6) We examined the relative intake of various macronutrients in relation to dementia, based on previous literature indicating potential associations with cognitive health [49–52].

## Results

Participant’s baseline characteristics at the time of dietary assessment are described in Tables 1a-1b for the overall cohort and stratified by quintile of Mediterranean or DASH diet score; quintiles are not uniform because everyone with the same diet score was categorized into the same quintile. At baseline, participants were a mean age of 50.6 (SD: 8.4) years; most were men (77.5%) and White (96.4%). Mediterranean and DASH diet scores were normally distributed; most participants reported a dietary pattern that corresponded to moderate adherence to either diet and few reported dietary patterns that corresponded to very low or high adherence (Appendix Figs. 1 and 2).

**Table 1a:** Characteristics of the study sample, overall and by Mediterranean diet score (n = 9,095)

Characteristic	Full study cohort	Quintiles of Mediterranean diet score^a^ (absolute score)				
		Q1 (0–2)	Q2 (3)	Q3 (4)	Q4 (5)	Q5 (6–9)
	n (%)	n (%)	n (%)	n (%)	n (%)	n (%)
N	9095 (100)	1445 (15.9)	1697 (18.7)	1963 (21.6)	1808 (19.9)	2182 (24.0)
Baseline age, mean (SD)	50.6 (8.4)	48.8 (7.9)	49.6 (8.0)	50.3 (8.4)	51.3 (8.5)	52.1 (8.6)
Male	7048 (77.5)	1202 (83.2)	1307 (77.0)	1508 (76.8)	1369 (75.7)	1662 (76.2)
Race or ethnicity						
White	8769 (96.4)	1396 (96.6)	1618 (95.3)	1894 (96.5)	1749 (96.7)	2112 (96.8)
Black	53 (0.6)	10 (0.7)	14 (0.8)	12 (0.6)	9 (0.5)	8 (0.4)
Hispanic	62 (0.7)	11 (0.8)	14 (0.8)	16 (0.8)	10 (0.6)	11 (0.5)
Other race or ethnicity	77 (0.8)	8 (0.6)	13 (0.8)	13 (0.7)	18 (1)	25 (1.1)
Missing	134 (1.5)	20 (1.4)	38 (2.2)	28 (1.4)	22 (1.2)	26 (1.2)
Bachelor’s degree or higher						
No	936 (10.3)	160 (11.1)	187 (11.0)	213 (10.9)	171 (9.5)	205 (9.4)
Yes	3094 (34.0)	493 (34.1)	576 (33.9)	665 (33.9)	585 (32.4)	775 (35.5)
Missing^b^	5065 (55.7)	792 (54.8)	934 (55.0)	1085 (55.3)	1052 (58.2)	1202 (55.1)
Current smoker						
No	8200 (90.2)	1229 (85.1)	1501 (88.5)	1783 (90.8)	1664 (92.0)	2023 (92.7)
Yes	895 (9.8)	216 (14.9)	196 (11.5)	180 (9.2)	144 (8.0)	159 (7.3)
	Mean (SD)	Mean (SD)	Mean (SD)	Mean (SD)	Mean (SD)	Mean (SD)
Maximal cardiorespiratory fitness (METs)^c^	11.4 (2.6)	11.1 (2.4)	11.2 (2.5)	11.4 (2.5)	11.4 (2.6)	11.8 (2.7)
Alcohol consumption (drinks per week)	5.4 (7.0)	4.8 (7.7)	5.2 (7.3)	5.0 (6.7)	5.3 (6.8)	6.2 (6.8)
BMI (kg/m^2^)	25.8 (3.9)	26.8 (4.1)	26.3 (4)	25.9 (3.9)	25.5 (3.7)	25.1 (3.6)
Dietary energy (kcal)	2134.2 (620.9)	2093.1 (617)	2079.6 (597.1)	2113.8 (623.2)	2130.9 (607.7)	2225.0 (640.9)
Fasting glucose (mg/dL)	99.8 (17.1)	101.1 (20.7)	100.0 (15.6)	99.6 (16.4)	99.4 (16.3)	99.5 (16.7)
Total cholesterol (mg/dL)	211.0 (39.1)	212.1 (39.8)	212.8 (39.6)	210.3 (38.2)	211.0 (38.2)	209.5 (39.7)
Systolic blood pressure (mm Hg)	121.0 (14.5)	120.6 (13.6)	120.9 (14.1)	120.4 (14.3)	121.2 (14.6)	121.6 (15.3)
Diastolic blood pressure (mm Hg)	80.9 (9.8)	81.4 (9.7)	81.1 (10)	80.5 (9.6)	80.9 (9.9)	80.6 (9.7)

*Abbreviations* BMI, body mass index; kcal, kilocalories; METs, metabolic equivalent of task; SD, standard deviation

^a^See the Methods for calculation of the Mediterranean diet score. Higher scores indicate greater adherence to the diet

^b^The maximum likelihood method [45] was used to include observations with missing data on education in regression analysis

^c^Cardiorespiratory fitness was expressed as maximal metabolic equivalents of task (METs) and assessed during the baseline clinic visit via a maximal treadmill test using a modified Balke protocol

**Table 1b:** Characteristics of the study sample, overall and by DASH diet score (n = 9,095)

Characteristic	Full study cohort	Quintiles of DASH diet score^a^ (absolute score)				
		Q1 (8–19)	Q2 (20–22)	Q3 (23–25)	Q4 (26–28)	Q5 (29–40)
	n (%)	n (%)	n (%)	n (%)	n (%)	n (%)
N	9095 (100)	1542 (17.0)	1939 (21.3)	2244 (24.7)	1781 (19.6)	1589 (17.5)
Baseline age, mean (SD)	50.6 (8.4)	48.4 (7.5)	49.5 (7.7)	50.5 (8.4)	51.6 (8.6)	52.8 (9.1)
Male	7048 (77.5)	1352 (87.7)	1600 (82.5)	1718 (76.6)	1285 (72.2)	1093 (68.8)
Race or ethnicity						
White	8769 (96.4)	1466 (95.1)	1875 (96.7)	2162 (96.3)	1721 (96.6)	1545 (97.2)
Black	53 (0.6)	16 (1.0)	15 (0.8)	14 (0.6)	4 (0.2)	4 (0.3)
Hispanic	62 (0.7)	13 (0.8)	17 (0.9)	9 (0.4)	13 (0.7)	10 (0.6)
Other race or ethnicity	77 (0.8)	14 (0.9)	13 (0.7)	16 (0.7)	20 (1.1)	14 (0.9)
Missing	134 (1.5)	33 (2.1)	19 (1.0)	43 (1.9)	23 (1.3)	16 (1.0)
Bachelor’s degree or higher						
No	936 (10.3)	178 (11.5)	195 (10.1)	216 (9.6)	186 (10.4)	161 (10.1)
Yes	3094 (34.0)	493 (32.0)	689 (35.5)	770 (34.3)	616 (34.6)	526 (33.1)
Missing^b^	5065 (55.7)	871 (56.5)	1055 (54.4)	1258 (56.1)	979 (55.0)	902 (56.8)
Current smoker						
No	8200 (90.2)	1268 (82.2)	1690 (87.2)	2031 (90.5)	1685 (94.6)	1526 (96)
Yes	895 (9.8)	274 (17.8)	249 (12.8)	213 (9.5)	96 (5.4)	63 (4.0)
	Mean (SD)	Mean (SD)	Mean (SD)	Mean (SD)	Mean (SD)	Mean (SD)
Maximal cardiorespiratory fitness (METs)^c^	11.4 (2.6)	10.9 (2.3)	11.2 (2.3)	11.4 (2.6)	11.6 (2.6)	12.0 (2.9)
Alcohol consumption (drinks per week)	5.4 (7.0)	6.0 (7.9)	5.9 (7.5)	5.3 (6.9)	5.1 (6.6)	4.5 (6.0)
BMI (kg/m^2^)	25.8 (3.9)	27.2 (4.1)	26.5 (3.9)	25.9 (3.9)	25.2 (3.5)	24.3 (3.5)
Dietary energy (kcal)	2134.2 (620.9)	2201.7 (637.5)	2145.9 (626.2)	2095.1 (622.3)	2085.2 (604.6)	2164.7 (606.2)
Fasting glucose (mg/dL)	99.8 (17.1)	101.2 (16.9)	101.2 (17.8)	99.8 (17.6)	99.1 (15.9)	97.9 (16.7)
Total cholesterol (mg/dL)	211.0 (39.1)	218.7 (40.9)	213.3 (38.1)	210.0 (39.1)	207.7 (38.5)	205.7 (37.7)
Systolic blood pressure (mm Hg)	121.0 (14.5)	121.8 (13.7)	121.0 (14.0)	120.8 (14.7)	120.0 (14.3)	121.6 (15.5)
Diastolic blood pressure (mm Hg)	80.9 (9.8)	82.3 (9.8)	81.3 (9.9)	80.7 (9.7)	80.1 (9.6)	80.0 (9.6)

*Abbreviations* BMI: body mass index; DASH: Dietary Approaches to Stop Hypertension; kcal: kilocalories; METs: metabolic equivalent of task; Q: quintile; SD: standard deviation

^a^See the Methods for calculation of the DASH diet score. Higher scores indicate greater adherence to the diet

^b^The maximum likelihood method [45] was used to include observations with missing data on education in regression analysis

^c^Cardiorespiratory fitness was expressed as maximal metabolic equivalents of task (METs) and assessed during the baseline clinic visit via a maximal treadmill test using a modified Balke protocol

The mean CRF level was 11.4 (SD: 2.6) METs; CRF levels increased as adherence to a Mediterranean or DASH diet increased (*p* < 0.001). Other indicators of a healthy lifestyle were also associated with Mediterranean or DASH diet adherence, including being a non-smoker, lower BMI, lower fasting glucose, and lower total cholesterol (all *p* ≤ 0.02).

The mean gap time between baseline clinic visit and Medicare enrollment was 15.9 (SD: 6.6) years. After a mean follow-up within Medicare of 9.2 (SD: 5.8) years, 1449 cases of all-cause dementia were identified (17.3 cases per 1,000 person-years). No violations of the proportional hazards assumption were observed. Neither Mediterranean diet adherence (fully adjusted HR per SD: 1.00, 95% CI: 0.94, 1.05) nor DASH diet adherence (fully adjusted HR per SD: 1.02, 95% CI: 0.96, 1.08) were associated with all-cause dementia in multivariable-adjusted models (with or without potentially mediating covariates) (Tables 2 and 3). Conversely, in models fully adjusted for main effects, higher CRF was associated with a lower hazard of all-cause dementia (HR, per MET increase, Mediterranean model: 0.95, 95% CI: 0.92, 0.98; HR, per MET increase, DASH model: 0.96, 95% CI: 0.92, 0.97). Results for CRF were similar in less adjusted models excluding potentially mediating pathways. No significant interactions between diet scores and CRF were observed, nor between diet scores and sex.

**Table 2:** Estimated all-cause dementia hazard ratios for Mediterranean diet score and cardiorespiratory fitness

	M1: Age, sex, exam year	M2: M1 + smoking, education	M3: M2 + CRF	M4: M3 + caloric intake, BMI	M5: glucose, cholesterol, SBP
	HR (95% CI)				
Mediterranean diet score, per SD of continuous score	0.97 (0.91, 1.03)	0.98 (0.92, 1.03)	1.00 (0.95, 1.05)	1.00 (0.94, 1.05)	1.00 (0.94, 1.05)
Maximal cardiorespiratory fitness, per MET^a^	--	--	0.94 (0.92, 0.97)	0.95 (0.92, 0.97)	0.95 (0.92, 0.98)

*Abbreviations* BMI: body mass index; CI: confidence interval; CRF:cardiorespiratory fitness; HR: hazard ratio; M: model; MET: metabolic equivalent of task; SBP: systolic blood pressure; SD: standard deviation

^a^Estimates for maximal cardiorespiratory fitness from models 1–2 were not estimated because it was not the primary exposure of interest

**Table 3:** Estimated all-cause dementia hazard ratios for DASH diet score and cardiorespiratory fitness

	M1: Age, sex, exam year	M2: M1 + smoking, education	M3: M2 + CRF	M4: M3 + caloric intake, BMI	M5: glucose, cholesterol, SBP
	HR (95% CI)				
DASH diet score, per SD of continuous score	0.95 (0.90, 1.01)	0.98 (0.93, 1.04)	1.02 (0.96, 1.08)	1.02 (0.96, 1.08)	1.02 (0.96, 1.08)
Maximal cardiorespiratory fitness, per MET^a^	--	--	0.94 (0.92, 0.96)	0.94 (0.92, 0.97)	0.95 (0.92, 0.97)

*Abbreviations* BMI: body mass index; CI: confidence interval; CRF: cardiorespiratory fitness; DASH: Dietary Approaches to Stop Hypertension; HR: hazard ratio; M: model; MET: metabolic equivalent of task; SBP: systolic blood pressure; SD: standard deviation

^a^Estimates for maximal cardiorespiratory fitness from models 1–2 were not estimated because it was not the primary exposure of interest

### Sensitivity analysis

Multiple sensitivity analyses were conducted to ensure that our results were robust to various analytic decisions. (1) Using the residual method [47] as an alternative analytic approach to account for total caloric intake, estimated HRs were consistent with our original approach (Appendix Table 2, and 3). (2) In analysis that excluded education from all models, estimated HRs remained nearly identical (Appendix Table 4, and 5). (3) We used a Chebyshev polynomial expansion to assess non-linear trends in diet scores. Hazard ratio plots revealed no statistically significant evidence of non-linearity (Appendix Figs. 3 and 4). We also examined diet score as quintiles and found no statistically significant associations with dementia (Appendix Table 6, and 7). (4) In analysis of the DASH diet that assumed all dairy consumed was high-fat instead of low-fat, no statistically significant associations were observed and estimates were nearly identical to our primary analysis (Appendix Table 8). (5) In analysis that excluded participants with an earliest indication of dementia at ages ≤ 67 years, estimated HRs for diet scores and CRF remained nearly identical. (6) No statistically significant associations were observed for any macronutrient, though participants in the highest quintile of percent energy from protein may be at increased risk of all-cause dementia (HR: 1.13, 95% CI: 0.98, 1.29) (Appendix Table 9).

## Discussion

In this cohort of apparently healthy middle-aged adults seeking preventive care, adherence to a Mediterranean or DASH diet was not associated with subsequent all-cause dementia. In contrast, estimated maximal CRF was protective against dementia, though CRF did not modify the association between diet and dementia. Using robust methods of exposure and outcome ascertainment, these data contribute additional evidence of a null association between Mediterranean or DASH diet adherence and dementia. With Medicare follow-up through 2019, this study also extends previous analyses within this cohort demonstrating the neuroprotective properties of CRF (previous follow-up through 2009) [17, 21], and provides novel data that diet and CRF had no interactive effect on dementia risk.

The suggested neuroprotective effects of the Mediterranean and DASH diets are hypothesized to occur through the increased intake of dietary components with antioxidant and anti-inflammatory properties and an improvement in vascular risk factor profiles, though the exact mechanisms are complex and not well-established in humans [11, 53–55]. Evidence from previous interventional and observational studies linking diet and dementia is suggestive but not conclusive. For instance, a systematic review of randomized controlled trials (RCTs) investigating the Mediterranean diet found protective effects within individual trials related to select cognitive outcomes, but no effects on dementia [54]. Meanwhile, recent meta-analyses of observational studies in older adults have found mixed associations (either null or protective) between the Mediterranean diet and dementia [12, 13]. Similarly, comprehensive reviews of the Mediterranean and DASH diets report protective associations for some domains of cognitive function, but scarce and inconsistent evidence on dementia [11, 14, 15, 56]. A hybrid of the Mediterranean-DASH diets specifically developed for neuroprotection and prevention of dementia—MIND (Mediterranean-DASH Intervention for Neurodegenerative Delay)—has shown similarly mixed results [57–59].

The persistent mixed evidence is likely due to the vast heterogeneity in study design, populations, and methodologies [11, 14, 15, 56, 60], including the baseline health status of study participants; methods of dietary assessment, including the use of validated dietary tools, dietary pattern scoring, and duration of diet adherence; and duration of follow-up to capture outcomes, given that dementia is primarily an illness of older age [61].

Additionally, given the complexity of the brain changes that occur with dementia [1] and its multifactorial risk factors [30, 62], single lifestyle behaviors may not confer similar neuroprotective effects for all individuals. Rather, the most effective interventions for dementia prevention may be multimodal, with implementation strategies tailored to diverse populations [11, 30, 55]. In our study, no interactive effects were observed between diet and CRF, though CRF did have a neuroprotective association, confirming findings from previous studies within this cohort [17, 21] and among Nordic populations [18–20].

Future interventions should emphasize the neuroprotective properties of CRF, but also incorporate other suggestive neuroprotective domains including diet, vascular risk management, and social interaction [63–67]. For instance, the two-year multidomain FINGER trial that included diet, exercise, cognitive training, and vascular risk monitoring improved overall cognition, executive functioning, and processing speed in an at-risk elderly population in Finland [63, 64]. Another trial among an at-risk U.S. population found that a DASH diet combined with exercise and caloric restriction improved executive function-memory-learning domains and psychomotor speed; and cognitive improvements were mediated, in part, by fitness [66]. Future research should continue to investigate the importance of specific dietary components or patterns on dementia risk; explore the necessary duration and intensity of multimodal risk-prevention interventions; and identify which populations such interventions offer the most neuroprotective benefit [56].

Our study’s findings are subject to a few key limitations. The majority of the study sample were White, male, college-educated, and all were enrolled in Medicare fee-for-service, which enhances internal validity but limits generalizability to other populations, particularly those that have higher rates of dementia [68]. Further, our sample included relatively few participants with the highest levels of adherence to the Mediterranean or DASH diets, which may have limited our statistical power to detect associations between high diet adherence and dementia. Indeed, the highest levels of adherence to the Mediterranean or DASH diet have shown protective associations against MCI and Alzheimer’s disease, while moderate adherence has not [10, 58]. We may not have captured all cases of dementia because of incomplete surveillance between the baseline clinic visit and Medicare enrollment. The mean gap time between clinic visit and Medicare enrollment was 15.9 years, though Medicare surveillance began at a mean age of 66.5 years—nearly two decades before the estimated mean age of dementia onset in the U.S [61]. —ensuring minimal missing outcome data before surveillance began. However, the mean attained age of the cohort was only 75.7 years, so dementia diagnoses that occurred later in life were not captured; this may result in an underestimate of the association between dietary pattern and dementia if a Mediterranean or DASH diet delays the onset of dementia. Additionally, dietary assessment at a single timepoint during midlife did not allow us to examine how duration of diet adherence, subsequent changes to dietary pattern (or other covariates) after baseline, or dietary intake during critical windows of development or disease pathogenesis may affect dementia risk [69]. We also had insufficient data on participants’ sleep, which is linked to both diet and dementia [70].

However, the use of the CCLS cohort linked to Medicare claims provided a large sample size with robust exposure and outcome ascertainment. The use of multi-day dietary records, as used in our study, are considered a gold standard when validating nutrient intake [71], and CRF estimated via maximal exercise testing is strongly correlated with directly measured VO_2max_ [41, 42]. Additionally, over a median follow-up within Medicare of 9.2 years, we identified nearly 1500 cases of all-cause dementia using a validated algorithm with 80% sensitivity [72] from the Medicare CCW [32]. We were able to adjust our analyses for various confounding variables (e.g., cardiovascular risk factors measured via laboratory testing and a standardized maximal graded exercise test protocol), and sensitivity analyses confirmed that our results were robust to alternative analytic strategies.

## Conclusions

Among a cohort of generally healthy, middle-aged adults seeking preventive care, adherence to a Mediterranean or DASH diet was not associated with subsequent all-cause dementia. However, as evidenced in our findings and in previous studies [17–21], CRF is protective and should be emphasized in multimodal lifestyle interventions that aim to decrease the risk of dementia.

## Electronic supplementary material

Below is the link to the electronic supplementary material.

Supplementary Material 1

Supplementary Material 2

## Data Availability

The Cooper Center Longitudinal Study data are not publicly available. A scientific data request may be submitted to The Cooper Institute’s Scientific Review Board Committee for review.

## Abbreviations

BMI: Body mass index
CCLS: Cooper Center Longitudinal Study
CCW: Chronic Conditions Warehouse
CRF: Cardiorespiratory fitness
MET: Metabolic equivalent of task

## References

[1] 2023 Alzheimer’s disease facts and Fig. 2023 Apr. Report No.: 1552–5279 (Electronic) 1552–5260 (Linking) Contract No.: 4.

[2] van Dyck CH, Swanson CJ, Aisen P, Bateman RJ, Chen C, Gee M, et al. Lecanemab in Early Alzheimer’s Disease. N Engl J Med. 2023;388(1):9–21. 36449413 10.1056/NEJMoa2212948

[3] U.S. Department of Health and Human Services. National Plan to Address Alzheimer’s Disease: 2021 Update. 2021.

[4] Norton S, Matthews FE, Barnes DE, Yaffe K, Brayne C. Potential for primary prevention of Alzheimer’s disease: an analysis of population-based data. Lancet Neurol. 2014;13(8):788–94. 25030513 10.1016/S1474-4422(14)70136-X

[5] Livingston G, Huntley J, Sommerlad A, Ames D, Ballard C, Banerjee S, et al. Dementia prevention, intervention, and care: 2020 report of the Lancet Commission. Lancet. 2020;396(10248):413–46. 32738937 10.1016/S0140-6736(20)30367-6 PMC7392084

[6] Appel LJ, Moore TJ, Obarzanek E, Vollmer WM, Svetkey LP, Sacks FM, et al. A clinical trial of the effects of dietary patterns on blood pressure. DASH Collaborative Research Group. N Engl J Med. 1997;336(16):1117–24. 9099655 10.1056/NEJM199704173361601

[7] Willett WC, Sacks F, Trichopoulou A, Drescher G, Ferro-Luzzi A, Helsing E, et al. Mediterranean diet pyramid: a cultural model for healthy eating. Am J Clin Nutr. 1995;61(6 Suppl):s1402–6. 10.1093/ajcn/61.6.1402S 7754995

[8] Guasch-Ferre M, Willett WC. The Mediterranean diet and health: a comprehensive overview. J Intern Med. 2021;290(3):549–66. 34423871 10.1111/joim.13333

[9] Morze J, Danielewicz A, Hoffmann G, Schwingshackl L. Diet Quality as assessed by the healthy eating index, alternate healthy eating Index, Dietary approaches to stop hypertension score, and Health outcomes: a second update of a systematic review and Meta-analysis of Cohort studies. J Acad Nutr Diet. 2020;120(12):1998–2031. e15. 33067162 10.1016/j.jand.2020.08.076

[10] World Health Organization. Risk reduction of cognitive decline and dementia: WHO guidelines. Geneva: World Health Organization; 2019. 31219687

[11] McGrattan AM, McGuinness B, McKinley MC, Kee F, Passmore P, Woodside JV, et al. Diet and inflammation in cognitive ageing and Alzheimer’s Disease. Curr Nutr Rep. 2019;8(2):53–65. 30949921 10.1007/s13668-019-0271-4 PMC6486891

[12] Coelho-Junior HJ, Trichopoulou A, Panza F. Cross-sectional and longitudinal associations between adherence to Mediterranean diet with physical performance and cognitive function in older adults: a systematic review and meta-analysis. Ageing Res Rev. 2021;70:101395. 34153553 10.1016/j.arr.2021.101395

[13] Nucci D, Sommariva A, Degoni LM, Gallo G, Mancarella M, Natarelli F, et al. Association between Mediterranean diet and dementia and Alzheimer disease: a systematic review with meta-analysis. Aging Clin Exp Res. 2024;36(1):77. 38519775 10.1007/s40520-024-02718-6 PMC10959819

[14] van den Brink AC, Brouwer-Brolsma EM, Berendsen AAM, van de Rest O. The Mediterranean, Dietary approaches to stop hypertension (DASH), and Mediterranean-DASH intervention for neurodegenerative Delay (MIND) diets are Associated with Less Cognitive decline and a lower risk of Alzheimer’s Disease-A Review. Adv Nutr. 2019;10(6):1040–65. 31209456 10.1093/advances/nmz054 PMC6855954

[15] Ellouze I, Sheffler J, Nagpal R, Arjmandi B. Dietary Patterns and Alzheimer’s Disease: An Updated Review Linking Nutrition to Neuroscience. Nutrients. 2023;15(14). 10.3390/nu15143204 PMC10384681 37513622

[16] Liu YH, Gao X, Na M, Kris-Etherton PM, Mitchell DC, Jensen GL. Dietary pattern, Diet Quality, and dementia: a systematic review and Meta-analysis of prospective cohort studies. J Alzheimers Dis. 2020;78(1):151–68. 32955461 10.3233/JAD-200499

[17] Defina LF, Willis BL, Radford NB, Gao A, Leonard D, Haskell WL, et al. The association between midlife cardiorespiratory fitness levels and later-life dementia: a cohort study. Ann Intern Med. 2013;158(3):162–8. 23381040 10.7326/0003-4819-158-3-201302050-00005 PMC3926646

[18] Horder H, Johansson L, Guo X, Grimby G, Kern S, Ostling S, et al. Midlife cardiovascular fitness and dementia: a 44-year longitudinal population study in women. Neurology. 2018;90(15):e1298–305. 29540588 10.1212/WNL.0000000000005290 PMC5894933

[19] Kurl S, Laukkanen JA, Lonnroos E, Remes AM, Soininen H. Cardiorespiratory fitness and risk of dementia: a prospective population-based cohort study. Age Ageing. 2018;47(4):611–4. 29718064 10.1093/ageing/afy060

[20] Tari AR, Nauman J, Zisko N, Skjellegrind HK, Bosnes I, Bergh S, et al. Temporal changes in cardiorespiratory fitness and risk of dementia incidence and mortality: a population-based prospective cohort study. Lancet Public Health. 2019;4(11):e565–74. 31677775 10.1016/S2468-2667(19)30183-5

[21] Gafni T, Weinstein G, Leonard D, Barlow CE, DeFina LF, Pettee Gabriel K, et al. Independent and joint associations of cardiorespiratory fitness and BMI with dementia risk: the Cooper Center Longitudinal Study. BMJ Open. 2023;13(12):e075571. 38086580 10.1136/bmjopen-2023-075571 PMC10729062

[22] Mi MY, Gajjar P, Walker ME, Miller P, Xanthakis V, Murthy VL, et al. Association of healthy dietary patterns and cardiorespiratory fitness in the community. Eur J Prev Cardiol. 2023;30(14):1450–61. 10.1093/eurjpc/zwad113 PMC10562138 37164358

[23] Leckie RL, Weinstein AM, Hodzic JC, Erickson KI. Potential moderators of physical activity on brain health. J Aging Res. 2012;2012:948981. 23304508 10.1155/2012/948981 PMC3523571

[24] Scarmeas N, Luchsinger JA, Schupf N, Brickman AM, Cosentino S, Tang MX, et al. Physical activity, diet, and risk of Alzheimer disease. JAMA. 2009;302(6):627–37. 19671904 10.1001/jama.2009.1144 PMC2765045

[25] Blondell SJ, Hammersley-Mather R, Veerman JL. Does physical activity prevent cognitive decline and dementia? A systematic review and meta-analysis of longitudinal studies. BMC Public Health. 2014;14:510. 24885250 10.1186/1471-2458-14-510 PMC4064273

[26] Erickson KI, Hillman C, Stillman CM, Ballard RM, Bloodgood B, Conroy DE, et al. Physical activity, cognition, and brain outcomes: a review of the 2018 physical activity guidelines. Med Sci Sports Exerc. 2019;51(6):1242–51. 31095081 10.1249/MSS.0000000000001936 PMC6527141

[27] Stephen R, Hongisto K, Solomon A, Lonnroos E. Physical activity and Alzheimer’s Disease: a systematic review. J Gerontol Biol Sci Med Sci. 2017;72(6):733–9. 10.1093/gerona/glw251 28049634

[28] Silsbury Z, Goldsmith R, Rushton A. Systematic review of the measurement properties of self-report physical activity questionnaires in healthy adult populations. BMJ Open. 2015;5(9):e008430. 26373402 10.1136/bmjopen-2015-008430 PMC4577932

[29] Williams PT. Physical fitness and activity as separate heart disease risk factors: a meta-analysis. Med Sci Sports Exerc. 2001;33(5):754–61. 11323544 10.1097/00005768-200105000-00012 PMC2821586

[30] Khemka S, Reddy A, Garcia RI, Jacobs M, Reddy RP, Roghani AK, et al. Role of diet and exercise in aging, Alzheimer’s disease, and other chronic diseases. Ageing Res Rev. 2023;91:102091. 37832608 10.1016/j.arr.2023.102091 PMC10842571

[31] Key MN, Szabo-Reed AN. Impact of Diet and Exercise interventions on Cognition and Brain Health in older adults: a narrative review. Nutrients. 2023;15(11). 10.3390/nu15112495 PMC10255782 37299458

[32] Centers for Medicare & Medicaid Services. Chronic Conditions Data Warehouse 2023 [ https://www2.ccwdata.org/web/guest/home/

[33] Finley CE, Barlow CE, Halton TL, Haskell WL. Glycemic index, glycemic load, and prevalence of the metabolic syndrome in the cooper center longitudinal study. J Am Diet Assoc. 2010;110(12):1820–9. 21111092 10.1016/j.jada.2010.09.016

[34] Brodney S, McPherson RS, Carpenter RS, Welten D, Blair SN. Nutrient intake of physically fit and unfit men and women. Med Sci Sports Exerc. 2001;33(3):459–67. 11252075 10.1097/00005768-200103000-00020

[35] U.S. Department of Agriculture. FoodData Central 2023 [ https://fdc.nal.usda.gov/

[36] Cook AJ, Friday JE. Pyramid servings database for USDA survey food codes version 2.0 2004 [ https://www.ars.usda.gov/research/publications/publication/?seqNo115=171530

[37] Shah NS, Leonard D, Finley CE, Rodriguez F, Sarraju A, Barlow CE, et al. Dietary patterns and long-term survival: a retrospective study of healthy primary care patients. Am J Med. 2018;131(1):48–55. 28860032 10.1016/j.amjmed.2017.08.010

[38] Trichopoulou A, Costacou T, Bamia C, Trichopoulos D. Adherence to a Mediterranean diet and survival in a Greek population. N Engl J Med. 2003;348(26):2599–608. 12826634 10.1056/NEJMoa025039

[39] Fung TT, Chiuve SE, McCullough ML, Rexrode KM, Logroscino G, Hu FB. Adherence to a DASH-style diet and risk of coronary heart disease and stroke in women. Arch Intern Med. 2008;168(7):713–20. 18413553 10.1001/archinte.168.7.713

[40] Willis BL, Morrow JR Jr., Jackson AW, Defina LF, Cooper KH. Secular change in cardiorespiratory fitness of men: Cooper Center Longitudinal Study. Med Sci Sports Exerc. 2011;43(11):2134–9. 21448076 10.1249/MSS.0b013e31821c00a7

[41] Pollock ML, Bohannon RL, Cooper KH, Ayres JJ, Ward A, White SR, et al. A comparative analysis of four protocols for maximal treadmill stress testing. Am Heart J. 1976;92(1):39–46. 961576 10.1016/s0002-8703(76)80401-2

[42] Pollock ML, Foster C, Schmidt D, Hellman C, Linnerud AC, Ward A. Comparative analysis of physiologic responses to three different maximal graded exercise test protocols in healthy women. Am Heart J. 1982;103(3):363–73. 7064770 10.1016/0002-8703(82)90275-7

[43] American College of Sports Medicine. ACSM’s guidelines for exercise testing and prescription. Lippincott Williams & Wilkins; 2013. 10.1249/JSR.0b013e31829a68cf 23851406

[44] Xu J, Kalbfleisch JD, Tai B. Statistical analysis of illness-death processes and semicompeting risks data. Biometrics. 2010;66(3):716–25. 19912171 10.1111/j.1541-0420.2009.01340.x

[45] Rubin DB. Inference and missing data. Biometrika. 1976;63(3):581–92.

[46] Wang X, Ma H, Li X, Heianza Y, Manson JE, Franco OH, et al. Association of Cardiovascular Health with Life Expectancy Free of Cardiovascular Disease, Diabetes, Cancer, and Dementia in UK adults. JAMA Intern Med. 2023;183(4):340–9. 36848126 10.1001/jamainternmed.2023.0015 PMC9972243

[47] Willett W, Stampfer MJ. Total energy intake: implications for epidemiologic analyses. Am J Epidemiol. 1986;124(1):17–27. 3521261 10.1093/oxfordjournals.aje.a114366

[48] Press WHF, Teukolsky BP, Vetterling SA. W.T. Numerical recipes in C—the art of scientific computing. New York: Cambridge University Press; 1988.

[49] Roberts RO, Roberts LA, Geda YE, Cha RH, Pankratz VS, O’Connor HM, et al. Relative intake of macronutrients impacts risk of mild cognitive impairment or dementia. J Alzheimers Dis. 2012;32(2):329–39. 22810099 10.3233/JAD-2012-120862 PMC3494735

[50] Muth AK, Park SQ. The impact of dietary macronutrient intake on cognitive function and the brain. Clin Nutr. 2021;40(6):3999–4010. 34139473 10.1016/j.clnu.2021.04.043

[51] Pase MP, Himali JJ, Beiser AS, Aparicio HJ, Satizabal CL, Vasan RS, et al. Sugar- and artificially sweetened beverages and the risks of Incident Stroke and Dementia: a prospective cohort study. Stroke. 2017;48(5):1139–46. 28428346 10.1161/STROKEAHA.116.016027 PMC5405737

[52] Mohan D, Yap KH, Reidpath D, Soh YC, McGrattan A, Stephan BCM, et al. Link between Dietary Sodium Intake, cognitive function, and Dementia Risk in Middle-aged and older adults: a systematic review. J Alzheimers Dis. 2020;76(4):1347–73. 32675410 10.3233/JAD-191339 PMC7504986

[53] Fotuhi M, Zandi PP, Hayden KM, Khachaturian AS, Szekely CA, Wengreen H, et al. Better cognitive performance in elderly taking antioxidant vitamins E and C supplements in combination with nonsteroidal anti-inflammatory drugs: the Cache County study. Alzheimers Dement. 2008;4(3):223–7. 18631971 10.1016/j.jalz.2008.01.004 PMC2684982

[54] Radd-Vagenas S, Duffy SL, Naismith SL, Brew BJ, Flood VM, Fiatarone Singh MA. Effect of the Mediterranean diet on cognition and brain morphology and function: a systematic review of randomized controlled trials. Am J Clin Nutr. 2018;107(3):389–404. 29566197 10.1093/ajcn/nqx070

[55] Kivipelto M, Mangialasche F, Ngandu T. Lifestyle interventions to prevent cognitive impairment, dementia and Alzheimer disease. Nat Rev Neurol. 2018;14(11):653–66. 30291317 10.1038/s41582-018-0070-3

[56] Scarmeas N, Anastasiou CA, Yannakoulia M. Nutrition and prevention of cognitive impairment. Lancet Neurol. 2018;17(11):1006–15. 30244829 10.1016/S1474-4422(18)30338-7

[57] Barnes LL, Dhana K, Liu X, Carey VJ, Ventrelle J, Johnson K, et al. Trial of the MIND Diet for Prevention of Cognitive decline in older persons. N Engl J Med. 2023;389(7):602–11. 37466280 10.1056/NEJMoa2302368 PMC10513737

[58] Morris MC, Tangney CC, Wang Y, Sacks FM, Bennett DA, Aggarwal NT. MIND diet associated with reduced incidence of Alzheimer’s disease. Alzheimers Dement. 2015;11(9):1007–14. 25681666 10.1016/j.jalz.2014.11.009 PMC4532650

[59] Chen H, Dhana K, Huang Y, Huang L, Tao Y, Liu X, et al. Association of the Mediterranean Dietary approaches to stop hypertension intervention for neurodegenerative Delay (MIND) Diet with the risk of Dementia. JAMA Psychiatry. 2023;80(6):630–8. 37133875 10.1001/jamapsychiatry.2023.0800 PMC10157510

[60] Morris MC. Nutrition and risk of dementia: overview and methodological issues. Ann N Y Acad Sci. 2016;1367(1):31–7. 27116239 10.1111/nyas.13047 PMC4849169

[61] Plassman BL, Langa KM, McCammon RJ, Fisher GG, Potter GG, Burke JR, et al. Incidence of dementia and cognitive impairment, not dementia in the United States. Ann Neurol. 2011;70(3):418–26. 21425187 10.1002/ana.22362 PMC3139807

[62] Kulmala J, Ngandu T, Kivipelto M. Prevention matters: Time for Global Action and effective implementation. J Alzheimers Dis. 2018;64(s1):S191–8. 29504541 10.3233/JAD-179919

[63] Ngandu T, Lehtisalo J, Solomon A, Levalahti E, Ahtiluoto S, Antikainen R, et al. A 2 year multidomain intervention of diet, exercise, cognitive training, and vascular risk monitoring versus control to prevent cognitive decline in at-risk elderly people (FINGER): a randomised controlled trial. Lancet. 2015;385(9984):2255–63. 25771249 10.1016/S0140-6736(15)60461-5

[64] Rosenberg A, Ngandu T, Rusanen M, Antikainen R, Backman L, Havulinna S, et al. Multidomain lifestyle intervention benefits a large elderly population at risk for cognitive decline and dementia regardless of baseline characteristics: the FINGER trial. Alzheimers Dement. 2018;14(3):263–70. 29055814 10.1016/j.jalz.2017.09.006

[65] Krakovska O, Christie GJ, Farzan F, Sixsmith A, Ester M, Moreno S. Healthy memory aging - the benefits of regular daily activities increase with age. Aging. 2021;13(24):25643–52. 34915450 10.18632/aging.203753 PMC8751597

[66] Smith PJ, Blumenthal JA, Babyak MA, Craighead L, Welsh-Bohmer KA, Browndyke JN, et al. Effects of the dietary approaches to stop hypertension diet, exercise, and caloric restriction on neurocognition in overweight adults with high blood pressure. Hypertension. 2010;55(6):1331–8. 20305128 10.1161/HYPERTENSIONAHA.109.146795 PMC2974436

[67] Blumenthal JA, Smith PJ, Mabe S, Hinderliter A, Lin PH, Liao L, et al. Lifestyle and neurocognition in older adults with cognitive impairments: a randomized trial. Neurology. 2019;92(3):e212–23. 30568005 10.1212/WNL.0000000000006784 PMC6340382

[68] Mather MSP. The demography of dementia and dementia caregiving. Washington, DC: Population Reference Bureau; 2020.

[69] Zhao C, Noble JM, Marder K, Hartman JS, Gu Y, Scarmeas N. Dietary patterns, physical activity, sleep, and risk for Dementia and Cognitive decline. Curr Nutr Rep. 2018;7(4):335–45. 30413973 10.1007/s13668-018-0247-9 PMC6905459

[70] Sabia S, Fayosse A, Dumurgier J, van Hees VT, Paquet C, Sommerlad A, et al. Association of sleep duration in middle and old age with incidence of dementia. Nat Commun. 2021;12(1):2289. 33879784 10.1038/s41467-021-22354-2 PMC8058039

[71] Yuan C, Spiegelman D, Rimm EB, Rosner BA, Stampfer MJ, Barnett JB, et al. Relative validity of nutrient intakes assessed by Questionnaire, 24-Hour recalls, and Diet Records as compared with urinary recovery and plasma concentration biomarkers: findings for women. Am J Epidemiol. 2018;187(5):1051–63. 29036411 10.1093/aje/kwx328 PMC5928456

[72] Taylor DH Jr., Fillenbaum GG, Ezell ME. The accuracy of medicare claims data in identifying Alzheimer’s disease. J Clin Epidemiol. 2002;55(9):929–37. 12393082 10.1016/s0895-4356(02)00452-3

