# Supplementary material for: Dietary patterns and cardiorespiratory fitness in midlife and subsequent all-cause dementia: findings from the Cooper Center Longitudinal Study
Source: Int J Behav Nutr Phys Act. 2024 Sep 27;21:109. doi: 10.1186/s12966-024-01663-x (PMC11428374; doi:10.1186/s12966-024-01663-x)
Supplement: Supplementary file 2 — Supplementary Material 2 [file 12966_2024_1663_MOESM2_ESM.docx]

Appendix Figure 1. Histogram of Mediterranean diet scores among the study sample


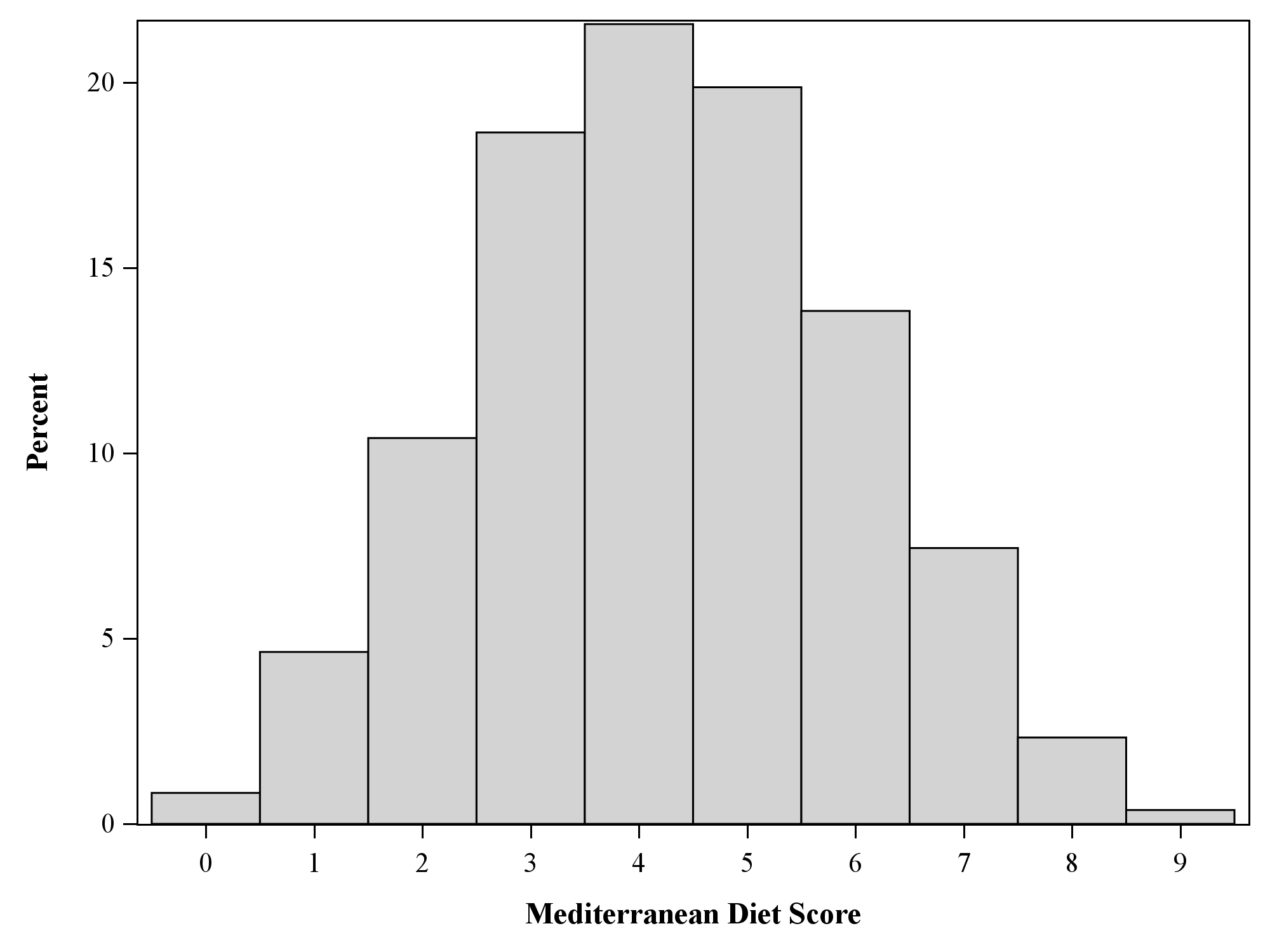


Appendix Figure 2. Histogram of Dietary Approaches to Stop Hypertension (DASH) diet scores among the study sample


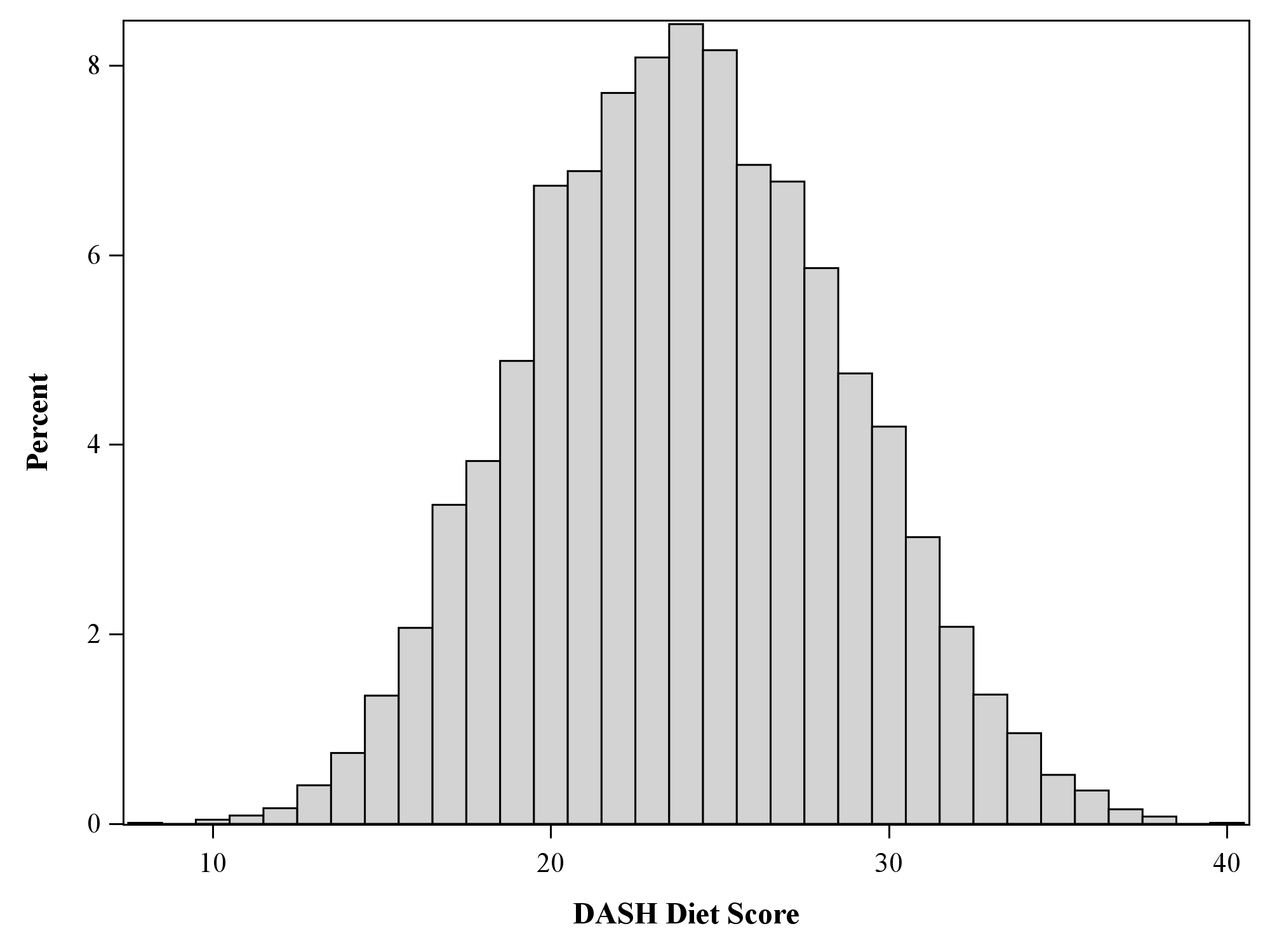


Appendix Figure 3. Nonlinear hazard ratio versus standardized Mediterranean diet score among the study sample


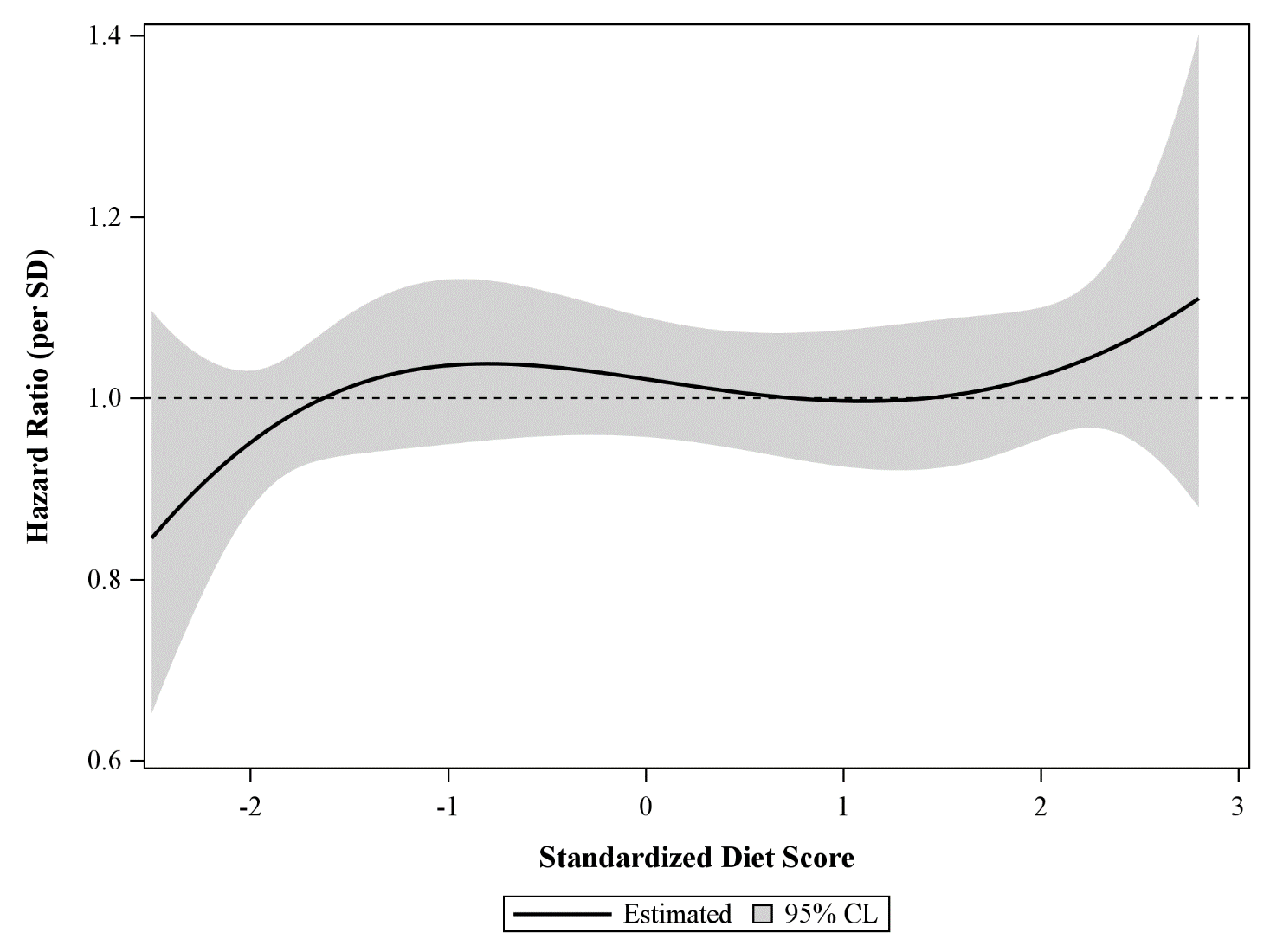


Footnote: The hazard ratio plot was produced using a Chebyshev polynomial expansion. The model adjusted for age, sex, clinic examination year, education, current smoking, and fitness (excluding potentially mediating pathways).

Appendix Figure 4. Nonlinear hazard ratio versus standardized Dietary Approaches to Stop Hypertension (DASH) diet score among the study sample


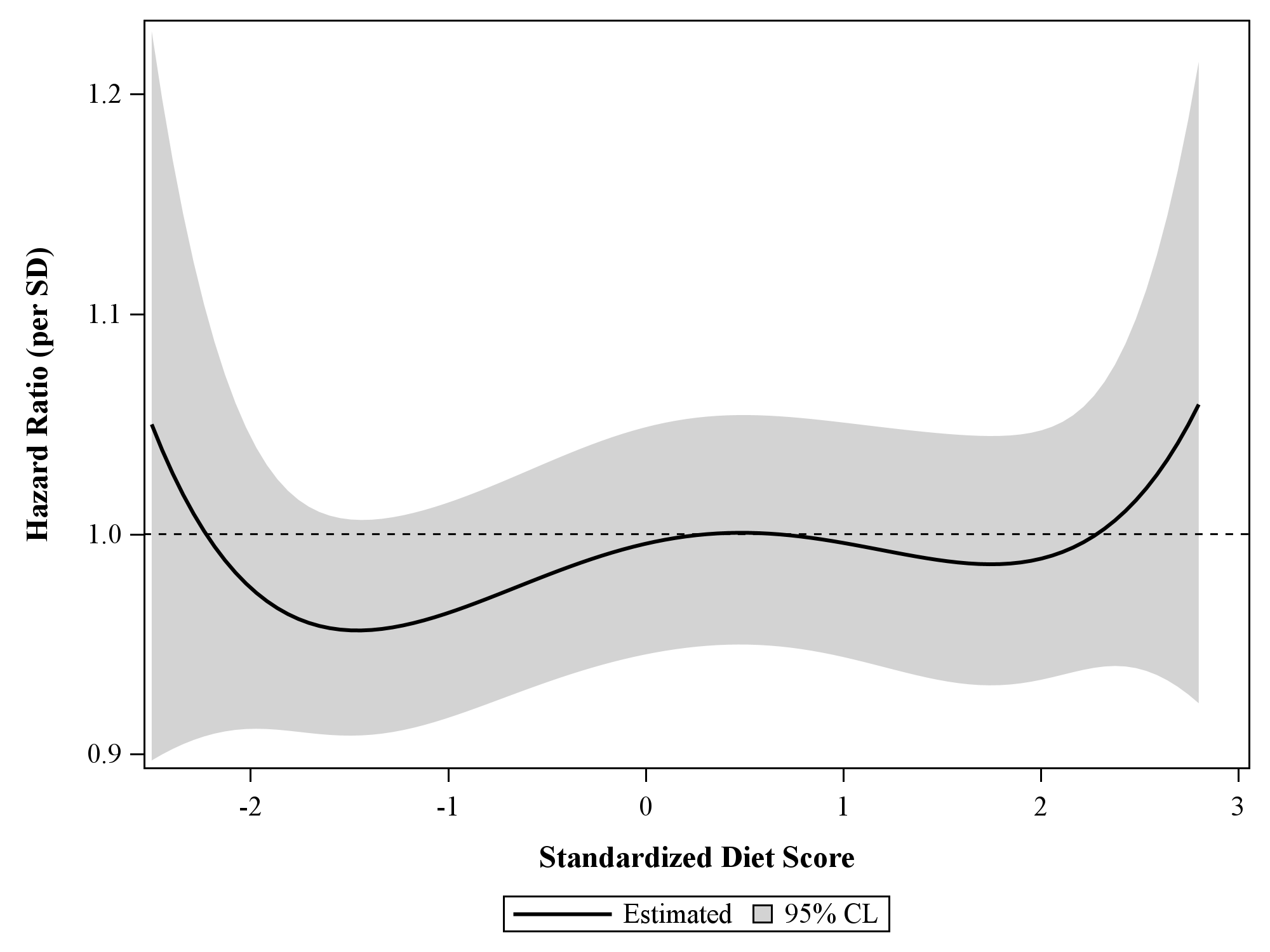


Footnote: The hazard ratio plot was produced using a Chebyshev polynomial expansion. The model adjusted for age, sex, clinic examination year, education, current smoking, and fitness (excluding potentially mediating pathways).

Appendix Table 1. Centers for Medicare & Medicaid Services Chronic Conditions Warehouse algorithm for identification of Alzheimer’s disease and related disorders or senile dementia

| Reference period | 3 years |
| --- | --- |
| Number/type of claims to qualify | At least 1 inpatient, skilled nursing facility, home health agency, hospital outpatient, or carrier claim with DX code |
| Valid ICD-10 codes | F01.50, F01.51, F02.80, F02.81, F03.90, F03.91, F04, F05, F06.1, F06.8, G13.8, G30.0, G30.1, G30.8, G30.9, G31.01, G31.09, G31.1, G31.2, G94, R41.81, R54 (any DX on the claim) |
| Valid ICD-9 codes | 331.0, 331.11, 331.19, 331.2, 331.7, 290.0, 290.10, 290.11, 290.12, 290.13, 290.20, 290.21, 290.3, 290.40, 290.41, 290.42, 290.43, 294.0, 294.10, 294.11, 294.20, 294.21, 294.8, 797 (any DX on the claim) |

Appendix Table 2. Estimated all-cause dementia hazard ratios for Mediterranean diet score and cardiorespiratory fitness, excluding educational status from all models^a^

|  | M1: Age, sex, exam year | M2: M1 + smoking | M3: M2 + fitness | M4: M3 + caloric intake, BMI | M5: glucose, cholesterol, SBP |
| --- | --- | --- | --- | --- | --- |
|  | HR (95% CI) | | | | |
| Mediterranean diet score, per SD of continuous score | 0.97  (0.91, 1.03) | 0.98  (0.92, 1.04) | 1.01  (0.95, 1.07) | 1.01  (0.94, 1.07) | 1.00  (0.94, 1.07) |
| Maximal cardiorespiratory fitness, per MET^b^ | -- | -- | 0.93  (0.90, 0.95) | 0.93  (0.90, 0.97) | 0.94  (0.91, 0.97) |

Abbreviations: BMI, body mass index; CI, confidence interval; HR, hazard ratio; M, model; MET, metabolic equivalent of task; SBP, systolic blood pressure; SD, standard deviation

^a^Education was missing for 56% of participants. Observations with missing data were included in primary analysis using the maximum likelihood method.

^b^Estimates for maximal cardiorespiratory fitness from models 1-2 were not estimated because it was not the primary exposure of interest.

Appendix Table 3. Estimated all-cause dementia hazard ratios for the Dietary Approaches to Stop Hypertension (DASH) diet score and cardiorespiratory fitness, excluding educational status from all models^a^

|  | M1: Age, sex, exam year | M2: M1 + smoking | M3: M2 + fitness | M4: M3 + caloric intake, BMI | M5: glucose, cholesterol, SBP |
| --- | --- | --- | --- | --- | --- |
|  | HR (95% CI) | | | | |
| DASH diet score, per SD of continuous score | 0.95  (0.90, 1.01) | 0.97  (0.91, 1.03) | 1.02  (0.95, 1.09) | 1.02  (0.96, 1.09) | 1.02  (0.96, 1.09) |
| Maximal cardiorespiratory fitness, per MET^b^ | -- | -- | 0.93  (0.90, 0.95) | 0.93  (0.90, 0.96) | 0.94  (0.90, 0.97) |

Abbreviations: BMI, body mass index; CI, confidence interval; HR, hazard ratio; M, model; MET, metabolic equivalent of task; SBP, systolic blood pressure; SD, standard deviation

^a^Education was missing for 56% of participants. Observations with missing data were included in primary analysis using the maximum likelihood method.

^b^Estimates for maximal cardiorespiratory fitness from models 1-2 were not estimated because it was not the primary exposure of interest.

Appendix Table 4. Estimated all-cause dementia hazard ratios for Mediterranean diet score (quintiles) and cardiorespiratory fitness

|  | M1: Age, sex, exam year | M2: M1 + smoking, education | M3: M2 + fitness | M4: M3 + caloric intake, BMI | M5: glucose, cholesterol, SBP |
| --- | --- | --- | --- | --- | --- |
|  | HR (95% CI) | | | | |
| Mediterranean diet score (ref: Q1, score of 0-2) |  |  |  |  |  |
| Q2 (score of 3) | 0.98  (0.81, 1.18) | 0.99  (0.81, 1.19) | 1.01  (0.83, 1.22) | 1.00  (0.82, 1.21) | 1.01  (0.83, 1.22) |
| Q3 (score of 4) | 0.95  (0.79, 1.14) | 0.96  (0.80, 1.16) | 1.00  (0.83, 1.20) | 0.99  (0.83, 1.19) | 1.00  (0.83, 1.20) |
| Q4 (score of 5) | 0.98  (0.81, 1.17) | 1.00  (0.83, 1.20) | 1.04  (0.87, 1.25) | 1.04  (0.86, 1.24) | 1.04  (0.87, 1.25) |
| Q5 (score of 6-9) | 0.87  (0.73, 1.04) | 0.89  (0.75, 1.06) | 0.96  (0.80, 1.15) | 0.95  (0.79, 1.14) | 0.96  (0.80, 1.14) |
| Maximal cardiorespiratory fitness, per MET^a^ | -- | -- | 0.94  (0.92, 0.97) | 0.95  (0.92, 0.98) | 0.95  (0.92, 0.98) |

Abbreviations: BMI, body mass index; CI, confidence interval; HR, hazard ratio; M, model; MET, metabolic equivalent of task; Q, quintile; SBP, systolic blood pressure

^a^Estimates for maximal cardiorespiratory fitness from models 1-2 were not estimated because it was not the primary exposure of interest.

Appendix Table 5. Estimated all-cause dementia hazard ratios for Dietary Approaches to Stop Hypertension (DASH) diet score (quintiles) and cardiorespiratory fitness

|  | M1: Age, sex, exam year | M2: M1 + smoking, education | M3: M2 + fitness | M4: M3 + caloric intake, BMI | M5: glucose, cholesterol, SBP |
| --- | --- | --- | --- | --- | --- |
|  | HR (95% CI) | | | | |
| DASH diet score (ref: Q1, score of 8-19) |  |  |  |  |  |
| Q2 (score of 20-22) | 0.98  (0.81, 1.20) | 1.00  (0.82, 1.22) | 1.05  (0.86, 1.28) | 1.05  (0.86, 1.28) | 1.05  (0.86, 1.28) |
| Q3 (score of 23-25) | 1.00  (0.83, 1.20) | 1.02  (0.85, 1.24) | 1.09  (0.90, 1.32) | 1.10  (0.91, 1.33) | 1.10  (0.91, 1.33) |
| Q4 (score of 26-28) | 0.99  (0.81, 1.20) | 1.02  (0.84, 1.24) | 1.12  (0.92, 1.36) | 1.13  (0.93, 1.38) | 1.13  (0.93, 1.38) |
| Q5 (score of 29-40) | 0.89  (0.73, 1.08) | 0.93  (0.76, 1.13) | 1.04  (0.85, 1.28) | 1.05  (0.86, 1.29) | 1.05  (0.86, 1.29) |
| Maximal cardiorespiratory fitness, per MET^a^ | -- | -- | 0.94  (0.92, 0.96) | 0.94  (0.92, 0.97) | 0.95  (0.92, 0.97) |

Abbreviations: BMI, body mass index; CI, confidence interval; HR, hazard ratio; M, model; MET, metabolic equivalent of task; Q, quintile; SBP, systolic blood pressure

^a^Estimates for maximal cardiorespiratory fitness from models 1-2 were not estimated because it was not the primary exposure of interest.

Appendix Table 6. Estimated all-cause dementia hazard ratios for the Dietary Approaches to Stop Hypertension (DASH) diet score and cardiorespiratory fitness, assuming that all dairy consumed was high-fat instead of low-fat

|  | M1: Age, sex, exam year | M2: M1 + smoking, education | M3: M2 + fitness | M4: M3 + caloric intake, BMI | M5: glucose, cholesterol, SBP |
| --- | --- | --- | --- | --- | --- |
|  | HR (95% CI) | | | | |
| DASH diet score^a^, per SD of continuous score | 0.96  (0.90, 1.02) | 0.98  (0.93, 1.04) | 1.02  (0.96, 1.08) | 1.02  (0.96, 1.08) | 1.02  (0.96, 1.08) |
| Maximal cardiorespiratory fitness, per MET^b^ | -- | -- | 0.94  (0.92, 0.96) | 0.94  (0.92, 0.97) | 0.95  (0.92, 0.97) |

Abbreviations: BMI, body mass index; CI, confidence interval; HR, hazard ratio; M, model; MET, metabolic equivalent of task; SBP, systolic blood pressure; SD, standard deviation

^a^New DASH diet scores ranged from 8-36 instead of 8-40.

^b^Estimates for maximal cardiorespiratory fitness from models 1-2 were not estimated because it was not the primary exposure of interest.

Appendix Table 7. Estimated all-cause dementia hazard ratios for the relative intake of macronutrients

|  | Adjusted HR^a^ (95% CI) |
| --- | --- |
| Highest quintile of % energy from protein (ref: Q1-Q4) | 1.13 (0.98, 1.29) |
| Highest quintile of % energy from carbohydrates (ref: Q1-Q4) | 1.00 (0.88, 1.15) |
| Highest quintile of % energy from fat (ref: Q1-Q4) | 0.89 (0.76, 1.04) |
| Highest quintile of % energy from added sugar (ref: Q1-Q4) | 1.06 (0.92, 1.22) |
| Highest quintile of saturated fat/total fat ratio (ref: Q1-Q4) | 1.08 (0.95, 1.24) |
| Highest quintile of sodium/potassium ratio (ref: Q1-Q4) | 0.94 (0.79, 1.11) |

Abbreviations: CI, confidence interval; HR, hazard ratio; Q, quintile

^a^Adjusted for all listed variables as well as age, sex, clinic examination year, current smoking, alcohol, education, and maximal cardiorespiratory fitness.
